# From Bubbles to Nanobubbles

**DOI:** 10.3390/nano11102592

**Published:** 2021-10-01

**Authors:** George Z. Kyzas, Athanasios C. Mitropoulos

**Affiliations:** Department of Chemistry, International Hellenic University, GR 65404 Kavala, Greece; kyzas@chem.ihu.gr

**Keywords:** dispersed-air, dissolved-air, electroflotation, wastewaters, effluents, particles

## Abstract

Nanobubbles are classified into surface and bulk. The main difference between them is that the former is immobile, whereas the latter is mobile. The existence of sNBs has already been proven by atomic force microscopy, but the existence of bNBs is still open to discussion; there are strong indications, however, of its existence. The longevity of NBs is a long-standing problem. Theories as to the stability of sNBs reside on their immobile nature, whereas for bNBs, the landscape is not clear at the moment. In this preliminary communication, we explore the possibility of stabilizing a bNB by Brownian motion. It is shown that a fractal walk under specific conditions may leave the size of the bubble invariant.

## 1. Introduction

If you would like to witness nature’s ability to create perfection, the best example is of a bubble. Nature not only knows how to make beautiful bubbles but also how to make them easily. In science, once the forces that are involved in the formation of a bubble were discovered, a whole new chapter of physical laws on surface thermodynamics emerged. To this end, some very notable scientists, such as Laplace, Plateau, Lord Rayleigh, Willard Gibbs, and many others, have left their mark on this area of science.

The first study was on soap bubbles [1] consisting of two surfaces, where each is tense or contractile. Balancing this pressure difference, Laplace obtained his famous law [2]:

(1) $$\Delta P=\frac{4\gamma }{R}$$

where *γ* is the surface tension, and R is the radius of the bubble. Equation (1) shows that the smaller the bubble, the greater the pressure inside it, i.e., pressure and curvature rise and fall together.

Liquids have skin, but different liquids have different skin strengths. Although to blow a soap bubble is easy, to blow a water bubble is not. This is because the elastic strength of clean water is much less than that of soap solution. While water bubbles in air are not possible, air bubbles in water are a common phenomenon, and their stability has been a long-standing problem for more than 70 years [3,4,5,6,7].

**Figure 1:**
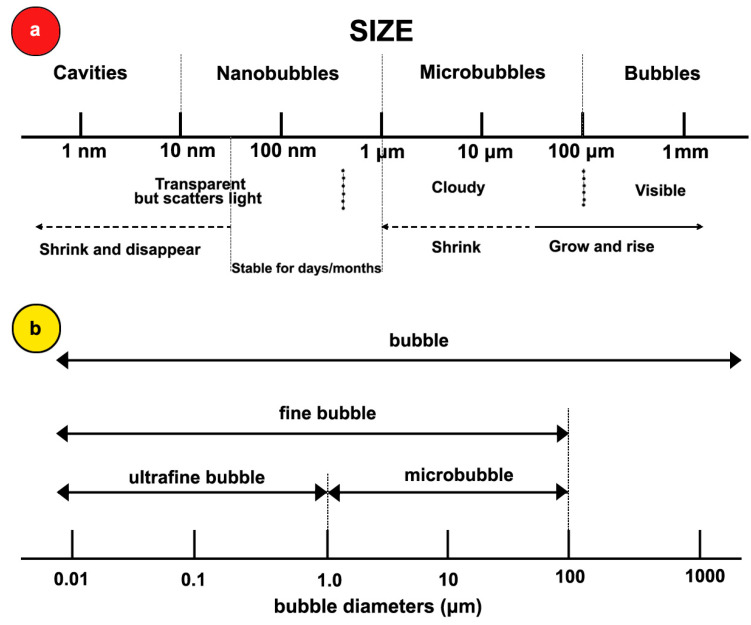
(**a**) The spectrum of bubble sizes according to their stability. (**b**) Bubble diameter classification according to ISO 20480-1-2017 [9].

In this study, we focus on nanobubbles (NBs), i.e., bubbles with sizes from 1 μm to 100 nm or less. According to diffusion theory, the lifetime of these bubbles ranges, respectively, from ms to μs. Therefore, they should not exist, but they do; not only do they exist, but they also persist almost indefinitely [8]. Figure 1a illustrates the whole bubble spectrum. According to ISO 20480-1-2017 [9], bulk NBs are also called ultrafine bubbles. Figure 1b shows this classification. The volume equivalent diameter *d_eq_* is given by the following equation:

(2) $$d_{eq}={\left(\frac{6V_{bubble}}{\pi }\right)}^{\frac{1}{3}}$$

where *V_bubble_* is the volume of the bubble, which also includes the bubble shell if the bubble is covered by a bubble shell. 

Early explanations on why NBs do not obey diffusion theory have concluded that this is due to the immobilization of the interface by surface contaminants [10] or to the impediment of the bubble by a solid particle known as the Harvey-nucleus [11,12,13]. Recent developments include further explanations such as hard hydrogen-bonding [14,15], electrostatic repulsion [16], dynamic equilibrium [17], or contact line pinning [18]; meanwhile, the debate goes on [19,20,21,22,23,24,25,26,27,28].

## 2. Historical Definition of the Problem

In a water column that is under negative pressure, a bubble may spontaneously appear within the liquid [29]. The process is known as cavitation, and the magnitude of the negative pressure at which it takes place defines the tensile strength of the liquid. In the case of homogeneous nucleation, the total energy required to form such a bubble is given by:

(3) $$\Delta E=4\pi R^{2}\gamma -\frac{4}{3}\pi R^{3}\Delta p=\frac{4}{3}\pi R^{2}\gamma$$

As the size of the nucleus grows, it reaches a critical (maximum) radius R_c_ just before the liquid raptures. At this point, the energy has a maximum value, ΔE_max_, which is formulated by Gibbs as:

(4) $$\Delta E_{\max}=\frac{16\pi \gamma^{3}}{3\Delta p_{c}},$$

where Δp_c_ is the pressure difference across the interface at the critical radius of the bubble. 

In 1850, Berthelot [30] measured the tensile strength of purified water to about 50 atm. On the other hand, the theoretical calculations of Frenkel [31] predicted a tension of 100 times the experimental one. Cavitation may, thus, be considered a precursor of NBs, for which the theory also fails to explain.

Let us denote with c_∞_ the gas concentration far away from the bubble and with c_s_ the gas concentration at the bubble–liquid interface. In an undersaturated solution, c_∞_ < c_s_, the NBs should dissolve away if the ambient pressure p_∞_ is sufficiently high. In an oversaturated solution, c_∞_ > c_s_, the NBs should grow, then rise and burst. In an even solution, c_∞_ = c_s_, the system is unstable, and the slightest disturbance will cause the bubble to either expand or to dissolve. Nevertheless, experience defies this prediction.

The mass transfer of gas in the liquid is governed by the diffusion equation [3]:

(5) $$\frac{\partial c}{\partial t}+\frac{dR}{dt}{\left(\frac{R}{r}\right)}^{2}\frac{\partial c}{\partial t}=\frac{D}{r^{2}}\frac{\partial }{\partial r}\left(r^{2}\frac{\partial c}{\partial r}\right),\;$$

where *c*(*r*,*t*) is the gas concentration in the liquid at time t and distance r from the center of a bubble of radius *R_o_* at *t* = 0, and *D* is the mass diffusivity. Since a bubble in a liquid has only one surface, the Laplace equation reduces by a factor of 2:

(6) $$\Delta p=\frac{2\gamma }{R}.$$

**Figure 2:**
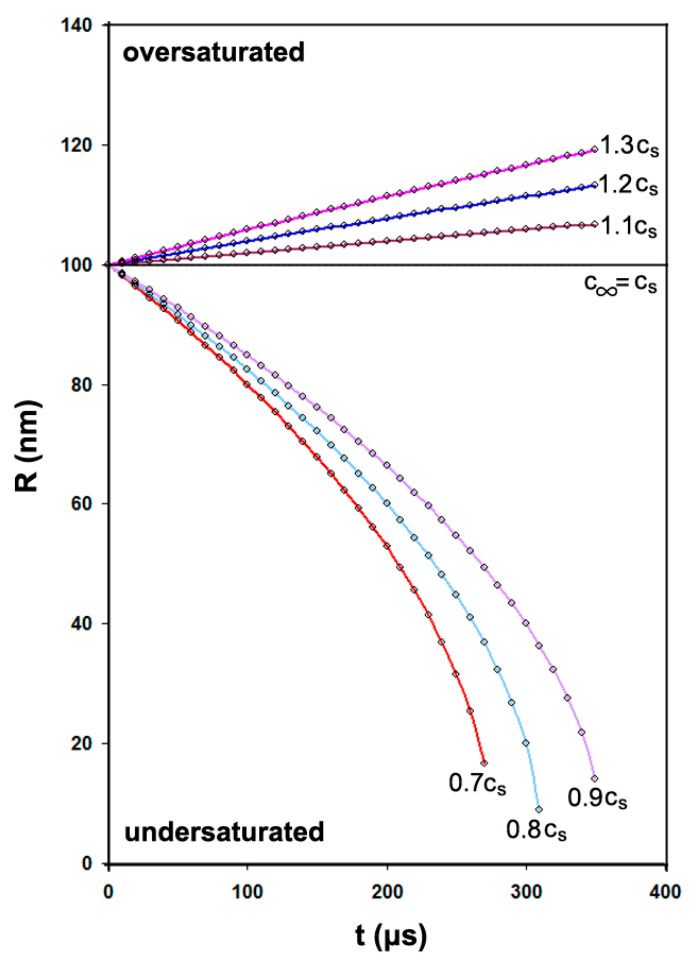
Shrinkage or growth of a bubble of radius 100 nm at various concentrations.

For a microbubble of R = 1 μm in water of γ = 72.8 mΝ/m, Δp = 1.5 bar, whereas for a nanobubble of 100 nm, Δp = 15 bar. According to Henry’s law:

(7) $$p=K_{H}c_{s},\;$$

where *p* is the pressure of the gas in the bubble, and *K_H_* is the Henry constant. By neglecting the second term on the left-hand side of Equation (5), Epstein and Plesset [4] found a solution that takes on the form:

(8) $$\psi^{2}=1\pm X^{2}\;\mathrm{with}\;\Psi =\frac{R}{R_{o}}\;\mathrm{and}\;X^{2}=\frac{2Dc_{s}}{\rho }\left(\frac{c_{\infty }}{c_{s}}-1\right)t,\;$$

where the (+) sign is for oversaturation (bubble expansion), the (−) sign is for undersaturation (bubble shrinkage), and ρ is the gas density. In the case of an air/water bubble at 295 K, *c_s_*/*ρ* = 0.02 and *D* = 2 × 10^−9^ m^2^/s. Figure 2 shows the growth and shrinkage of an NB of *R_o_* = 100 nm, and Table 1 records the fate of different size bubbles according to this theory. 

A perturbation as small as 0.01% on the equilibrium concentration will cause a bubble of radius 1 μm to dissolve in 2 min or to grow, without bounds, 10 times its original size in 3.4 h.

## 3. Explanations for NB Longevity

Fox and Herzfeld [10] have suggested that organic impurities (e.g., fatty acids) are adsorbed on the surface of the bubble and, as it contracts, will cover it completely with an organic skin that will slow down its contraction, although it will not reduce it to zero. A thin square sheet of area A_o_ under a uniform side force F will cause a relative change of that area by the following calculation [32]:

(9) $$\frac{A}{A_{o}}=\frac{Y\delta }{1-\nu }F,\;$$

where *Y* is the Young modulus, *δ* is the thickness of the film, and *ν* is Poisson’s constant. For a tearing strength of 50 mN/m and *δ* = 15 Å, one gets a strength of 33 bar. However, later on, Herzfeld by himself [33], based on experimental data [34], abandoned the rigid skin hypothesis.

Instead of an organic skin, Akulichev [35] proposed an ionic skin based on the fact that air/water bubbles possess an electrical charge [36]. Hydrophobic ions such as Cl^−^ will migrate to the surface of the bubble, whereas hydrophilic ones such as OH^−^ will not.

Sirotyuk [37] extended Fox and Herzfeld theory by suggesting stabilization due to a film of surface-active agents. In the case of air/water NBs, the polar head will bond to the water, and the tail will dangle in the air. This skin now has greater elasticity and will help the bubble to stabilize. Only traces of surface-active agents are needed to save them.

**Figure 3:**
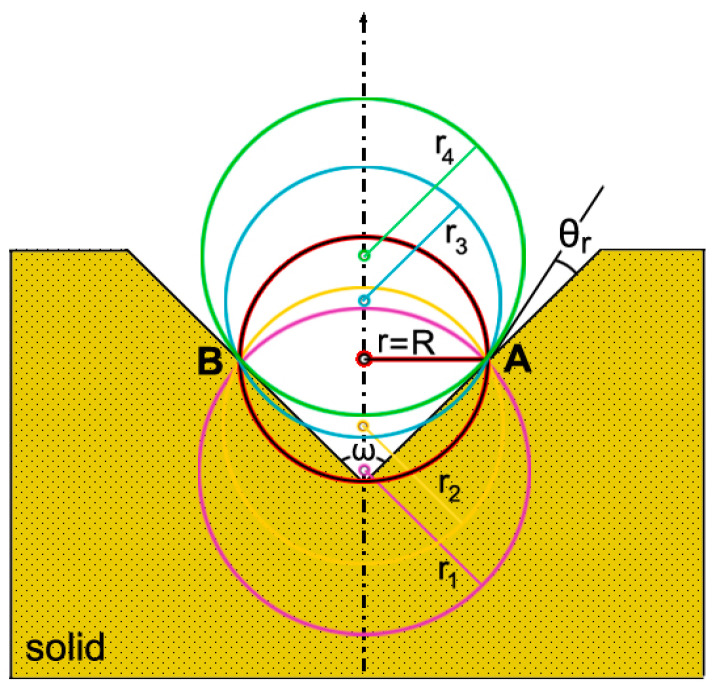
A bubble impended in a crevice. The course of events Δp increases, where r is the radius of curvature, θr is the receding angle (small), and R is the pinning half distance between Points A and B. When r = R, the bubble is hemispherical.

Harvey and coworkers [11] studied bubble formation in animals. They considered a solid impurity with a crevice containing gas. When the receding angle *θ_r_* is small, by increasing the pressure Δp across the gas–liquid interface, the radius of curvature decreases until r = R. Now, the bubble is unstable, and the slightest increase in Δp will cause the bubble to grow and the radius of curvature to increase. When the contact angle θ = θ_r_, the bubble will move up the side of the cavity until the buoyant force becomes greater than 2πR*γ* and burst. Figure 3 shows this course of events, where: r_1_ > r_2_ > r = R < r_3_ < r_4_. If, on the other hand, *θ_r_* is large enough, when θ_r_ = θ, the bubble will grow unpinned and eventually will move out of the cavity, rise, and burst. In both cases, a mass of gas may be left behind. 

**Figure 4:**
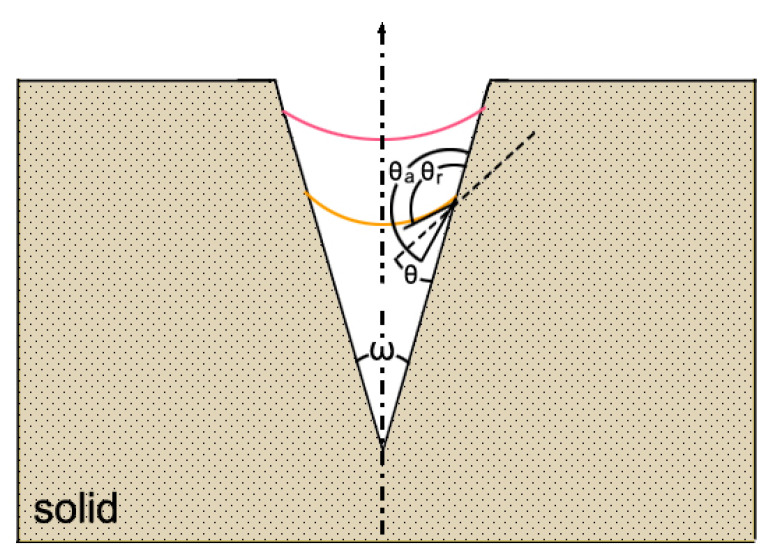
A bubble impended in a cone crevice of a very small apical angle, ω, and θ, the equilibrium contact angle, with θ_a_ as the advancing angle and θ_r_ the receding angle. Notice that the meniscus is now concave.

In order for the gas bubble to survive, the interface must be concave toward the liquid. This can be realized if the crevice is a cone with an acute apical angle, *ω*, so that: *c_a_* > (*π* + *ω*)/2, where *c_a_* is the advancing angle. Here, gas is creeping up the side of the cone until *θ* = *θ_r_*. The condition for equilibrium is now Δp = −2*γ*/R, where the negative sign indicates a concave curvature. Figure 4 illustrates this mechanism.

Recent advancements on NBs classify them to bulk and surface, with the main difference being the lack of a three-phase contact line for the former compared to the latter [38]. As a result, bulk NBs are mobile, whereas the surface is not, and, again, the radius of the curvature of bulk NBs is much smaller than that of surface NBs. Furthermore, the existence of sNBs has already been proven by atomic force microscopy (AFM) [39,40,41], while the existence of bNBs is indicative but not secured [14,42,43,44,45]. This doubt is due to the lack of an appropriate experimental method; dynamic light scattering (DLS), which is the prime experimental technique for detecting bNBs, cannot distinguish between bubbles, droplets, or particles [46]. However, the pursue of new techniques in determining both surface and bulk NBs is of vital importance for the advancement of NB technologies and their applications. To this end, Pan et al. [47] have investigated O_2_ bubbles of >25 nm at a diatomite particle in situ with synchrotron-based scanning transmission soft X-ray microscopy (STXM). They strongly indicate that in situ studies provide useful information on material preparation, phase equilibrium, nucleation kinetics, and chemical composition in the confined space. 

**Figure 5:**
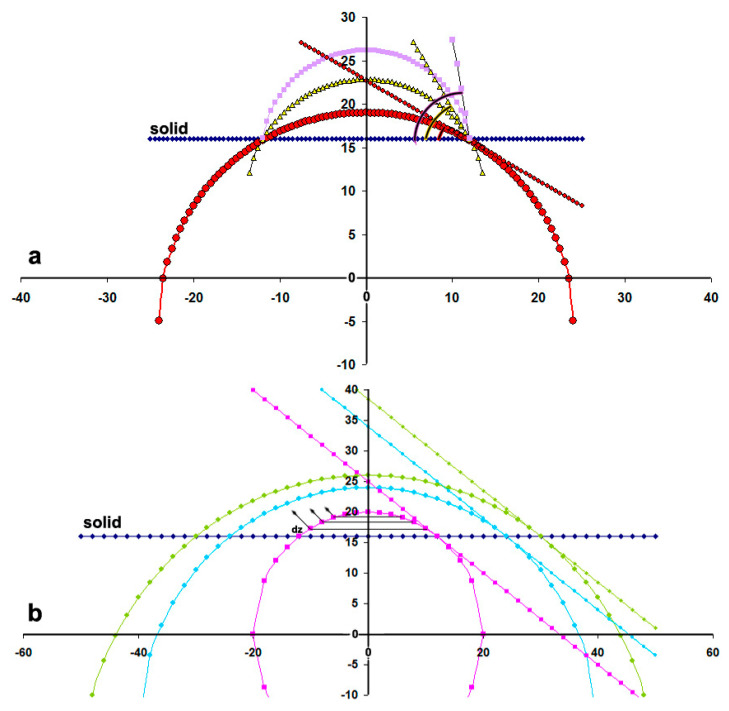
(**a**) Stabilization of an NB pinned on a surface. As the height and the contact angle decrease, the radius of the curvature increases and the pressure across the interface deflates. (**b**) Free-standing sNBs. Notice the general case of non-homogeneous distribution of the dissolved gas. By dividing the bubble into slices of thickness (dz) and by ignoring the gas concentration in the liquid, the bottom slices show the largest contribution to gas exchange according to Equation (12).

The maximum work W_c_ required for the formation of a surface bubble [48] of critical size is given by an equation similar to Equation (4), but with a factor Φ:

(10) $$W_{c}=\frac{16\pi \gamma^{3}}{3\Delta p_{c}}\Phi \;\mathrm{with}\;\Phi =\frac{1}{4}\left(2+\cos\theta_{c}\right)\times {\left(1-\cos\theta_{c}\right)}^{2},$$

where *θ_c_* is the critical contact angle from the side of gas. Based on this geometry, Brenner and Lohse [17] have introduced a dynamic equilibrium mechanism for sNB stabilization, where the gas out-flux *J_o_*_ut_ is compensated by gas in-flux *J_in_* at the contact line.

(11) $$J_{out}=\pi RD\left(1-\frac{c_{\infty }}{c_{s}}\right)=\frac{2\pi sDR}{\tan\theta },$$

where *s* > 0 is the attraction potential of gas from the hydrophobic wall, *θ* is the contact angle, and R is now the radius of the spherical cap circle. However, this model does not fit the experimental results, and some years later, Lohse and Zhang [49] suggested that pinning and gas oversaturation may explain the stability of sNBs. When the bubble shrinks, the radius of the curvature increases; therefore, Δp decreases and eventually becomes too weak to press out the bubble against the oversaturation. Figure 5a simulates this mechanism; for comparison, Figure 5b shows the case of free-standing sNBs.

It is noted, however, that experiments show that sNBs survive in open systems and undersaturated environments too. Based on the AFM images, Qian et al. [50] have argued that pinning, although good for the stabilization process, is not enough; it has to be accompanied by another effect to explain the observed stability. They suggested that a kinetic barrier may prevent the rapid transfer of gas across the interface of the sNB. Tan et al. proposed that in an undersaturated solution, the sNB would be stabilized by both pinning and attractive hydrophobic forces. By dividing the bubble into slices of thickness (dz; see Figure 5b), they developed the following equation for a localized concentration buildup, c(z):

(12) $$c(z)=c_{\infty }\exp\left(-\frac{\phi_{o}e^{-\frac{z}{\lambda }}}{k_{B}T}\right)$$

where *ϕ_ο_e^−z^*^/*λ*^ is the short-ranged potential, which is attractive when *ϕ_o_* < 0 and repulsive when *ϕ_o_* > 0; λ = 1 nm is the interaction distance. Equation (12) predicts either a localized oversaturation next to a hydrophobic substrate or a localized undersaturation next to a hydrophilic solid.

Based on IR spectra for O-H, Ohgaki et al. [14] have suggested that hydrogen bonding on the interface of a bNB is similar to that of gas hydrates. This hard hydrogen bonding helps the interface to resist the diffusivity of the gas from the bNB and to maintain a kinetic balance against the internal pressure.

**Figure 6:**
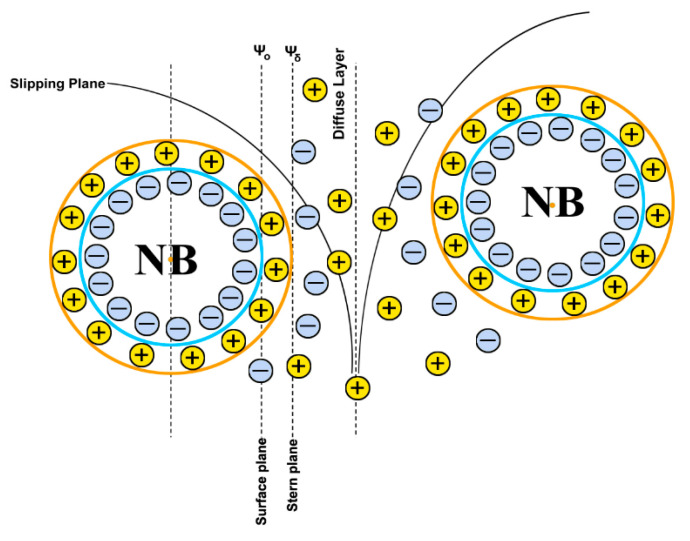
The electrical double layer for bNBs; Ψ_δ_ is the potential at the boundary between the compact and diffuse layers.

Experimental results have confirmed that NBs in water are negatively charged [16]. The zeta potential is about ζ = −40 mV in pure water (pH = 7). However, different values for different water purifications have been observed too. For instance, air NBs in deionized water have ζ = −65 mV, whereas in distilled water, ζ = −35 mV. Electrostatic repulsion may be added to the Laplace equation in a way that:

(13) $$\Delta P=\frac{2\gamma }{R}-\frac{\varepsilon \zeta^{2}}{R^{2}},\;$$

where *ε* is the dielectric constant. However, the second term of the RHS of Equation (13) is small to balance the Laplace pressure. Zang et al. and others [51,52] have proposed a correction for surface tension at higher pressures:

(14) $$\gamma =\gamma_{0}{\left(1-\frac{\rho }{\rho_{w}}\right)}^{4},\;$$

where *ρ_w_* = 1000 kg/m^3^ is the water density. In the case of an air bubble of *R* = 100 nm and *ρ* = 20 kg/m^3^, *γ* = 67 mN/m. However, even this correction does not qualify Equation (13) as a solution to the problem. Nevertheless, the negative charge of bNB surfaces will prevent them from coalescing and, again, will form an electric double layer [53]. These electrokinetic properties of bNBs may help them to survive for a longer time. Moreover, the many bNBs presented in the solution may also help their stabilization because the large concentrations of bNBs can supply gas to the liquid, retarding their dissolution [54]. Figure 6 illustrates the electrical double layer for bNBs.

## 4. Discussion

The NB survival problem is very old and very complex, one reason being that the phenomenon is not static but dynamic. Epstein and Plesset, in their seminal paper on the stability of gas bubbles in liquid–gas solutions, have made several assumptions. For instance, the transport term of the diffusion equation has been omitted, as well as the vapor pressure (only the gas pressure is considered). Again, the content of the bubble, the temperature, and the pressure inside it are assumed to be homogeneous and uniform, and, of course, the shape of the bubble is understood to be spherical. In the solution, the pressure far away from the bubble, the liquid density, and the viscosity are taken to be constant and uniform and the liquid to be incompressible. Although these assumptions are shown to be valid for bubbles as small as 1 μm [55], they may not be justified in the case of smaller ones. 

**Figure 7:**
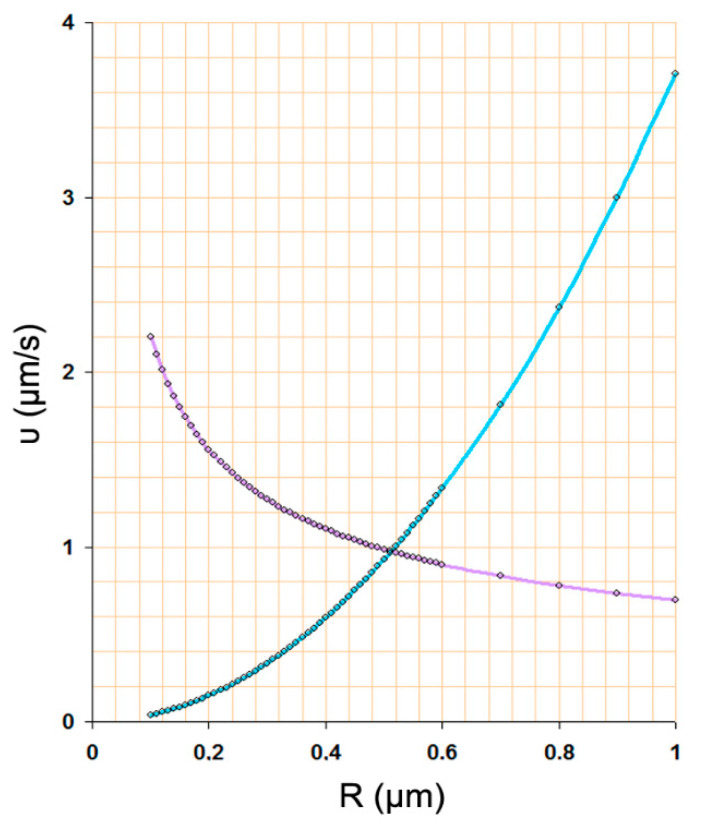
Buoyancy rise (cyan line) versus Brownian diffusion (purple line) for bubbles of different sizes. Cross-over point at R = 500 nm and υ = 1 μm/s.

Bubbles in a solution are subject to different kinds of motion; buoyancy rise and Brownian motion are of interest here. The terminal velocity υ_b_ due to buoyancy, balanced by the Rybczynski approximation, equals to [56]:

(15) $$\upsilon_{b}=\frac{g\rho_{w}}{3\eta }R^{2},$$

where *g* is the acceleration of gravity and *η* is the water viscosity. On the other hand, the mean velocity *υ_B_* due to Brownian motion is given by Einstein’s formula:

(16) $${\left[\frac{\langle x^{2}\rangle }{t^{2}}\right]}^{\frac{1}{2}}=\sqrt{\frac{2D}{t}}=\sqrt{\frac{R_{G}T}{3\pi \eta {Ν}_{A}Rt}}=\upsilon_{B},\;$$

where <*x*^2^> is the average particle displacement, *R_G_* is the gas constant, *N_A_* is Avogadro’s number, and *T* is the absolute temperature. Apparently, as t approaches 0, Equation (16) diverges and, therefore, does not represent the real velocity [57,58]. However, the smaller the bubble, the greater the velocity. Figure 7 compares the two given velocities for air/water bubbles of different radii. 

The cross size of the bubbles is ~1 μm. At this point, the average velocity is about 1 μm/s. For smaller bubbles, the Brownian motion dominates, and for larger ones, buoyancy prevails. In a saturated solution, a disturbance in the concentration of c_∞_–c_s_ = 0.01% will require 4 s for a 100 nm bubble to double its radius. At the same time, this doubled size bubble will be displaced ~6 μm away from its original position; that is, 60 times its original radius. Although the gas concentration in the bulk is taken to be spherically symmetric and the density in the bubble uniform, the motion of the bubble may put them in doubt. 

Nour nanoparticle tracking technique combines Brownian motion and light scattering to determine the bNBs’ diffusion constant and then to predict their spherical hydrodynamic diameter [59]. The moving bubble may not be spherical. Molecular dynamic simulations also point to the same conclusion [38]. As a result, the density of the gas within the bubble may not be uniform. Under Brownian motion, a bNB in a liquid, besides its inertia, will also be influenced by the inertia of the surrounding liquid. At short time scales (e.g., ns), such motion will also cause long-lived vortices in the liquid [57]. 

Another important physicochemical difference [60] between bulk and surface NBs is the Knudsen number, Kn. For a bNB, the Kn is always less than one: Kn = λ/R, where λ is the mean free path of the gas molecule. Therefore, the collision of gas molecules between each other dominates. The opposite behavior is common for sNBs, Kn = λ/h > 1, where h is the height of the spherical cap [61]. In this case, the collision of gas molecules with the bubble walls prevails. By excluding contamination as a universal mechanism for NB longevity of both bulk and surface, the solution to the problem must reside on their main difference, that is, the mobility of the former and immobility of the latter. Zou et al. [62] have studied bNBs in deionized water. They concluded that the bubbles remained stable for about two months due to Brownian motion. However, it must be noted that although several studies [63,64] have indicated that bNBs experience Brownian motion, the determination of the exact mechanism is still intriguing. 

**Figure 8:**
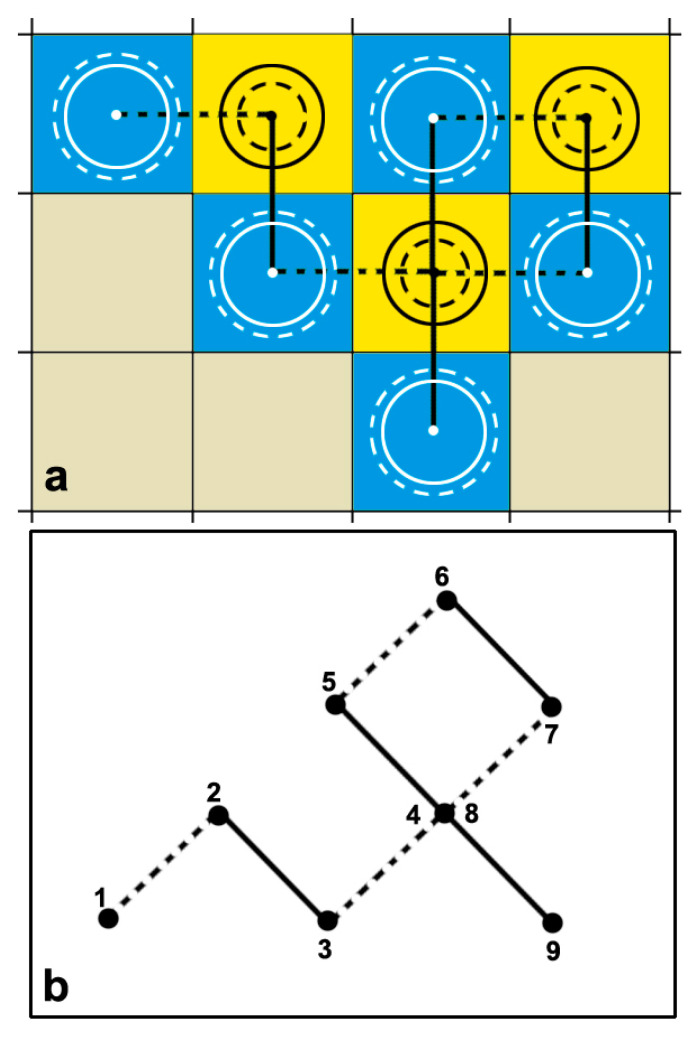
(**a**) A schematic representation of a bulk NB displacement, alternating from an oversaturated domain (blue) to an undersaturated domain (yellow) at equal times but with opposite perturbation conditions. Solid circles reflect the original size of the bubble, and broken circles indicate either expansion or contraction. Notice that the size of the average bubble is invariant. (**b**) The bubble performs a Brownian walk of an alternating Peano curve of fractal dimension H = 2.

In an oversaturated solution, when the bubble is stationary, the size of the bubble increases and the Laplace pressure decreases, causing a reduction in gas concentration in the vicinity of the bubble. As a result, more gas diffuses from the solution into the bubble, thus establishing a positive feedback loop. The opposite takes place when starting from an undersaturated solution. If we now assume that the solution is in an even situation, the slightest perturbation, over or under, will initiate one of the aforementioned processes, and the bubble either will grow and burst or shrink and dissolve. However, in the domain of the solution, since such a perturbation in another domain must be an opposite one, this thus establishes a long wave of perturbations that cover up all the extend of the solution. If the bubble is now from an oversaturated domain displaced by Brownian motion, which is known to be persistent to an undersaturated domain and so on, it has a chance to survive. Figure 8 illustrates a schematic representation of this perception.

In Equation (8), by assuming the growth of the bubble and then shrinkage of the expanded bubble under the same but opposite conditions and for equal time, we get:

(17) $$\begin{array}{l} R_{+}^{2}-R_{o}^{2}=+\frac{2Dc_{s}}{\rho }\left(\frac{c_{\infty }}{c_{s}}-1\right)t\\ R_{-}^{2}-R_{+}^{2}=-\frac{2Dc_{s}}{\rho }\left(\frac{c_{\infty }}{c_{s}}-1\right)t \end{array}\}\Rightarrow R_{o}^{2}-R_{+}^{2}=R_{-}^{2}-R_{+}^{2}\Rightarrow R_{o}^{2}=R_{-}^{2}$$

where *R*_+_ and *R*_−_ are the radii of the expanded and contracted bubbles, respectively. As a result, the average size of the bubble remains invariant. The displacements of the bubble in Figure 8a have a pattern shown in Figure 8b:

(18) $$\langle x_{+}^{2}\rangle +\langle x_{-}^{2}\rangle =\langle x^{2}\rangle ,$$

where <*x*_+_^2^> and <*x*_−_^2^> are the average displacements from over- to under-saturation. Hence, the generator of this pattern is made by two equal intervals of an angle of 90° alternating between the right and left of the teragon. This is a Peano curve with a fractal dimension H = 2 [65], thus connecting classical theory with the fractal character of Brownian motion [66,67].

## 5. Conclusions

Although there is an intense debate on the fundamentals of NB survival, there has not been equal hesitation from the industry on their usefulness. Applications to biology, water cleaning, the food industry, agriculture and hydroponics, and drug delivery ultrasonography are only some of the examples of using NBs successfully [68,69,70,71,72,73]. The Fine Bubble Industries Association (FBIA) and the Wall Street Journal have shown the business growth of NBs to have been from USD 20 million in 2010 to USD 10 billion in 2020. In the EU, the business is expected to grow from EUR 72 million in 2020 to EUR 145 million in 2030, where 52% of the market will be in the water treatment sector [46,74,75]. In this introductory note, we hope to ignite a discussion of new theories and radical hypotheses explaining how NBs avoid the Laplace catastrophe. Intuitively, it is understood that bubbles less than 1 μm in size may survive much longer than larger ones. At this size, diffusive Brownian motion crosses with buoyancy. Once a bubble is in the former region, it has a chance to survive for a longer time, while in the latter, it has not.

## Figures and Tables

**Table 1:** Dissolution and expansion of different size bubbles at various concentrations.

*R_o_*	c_∞_ < c_s_	Time to R = 0	c_∞_ > c_s_	Time to R = 10 *R_o_*
10 μm	0.75 c_s_	5 s	1.25 c_s_	495 s
1 μm		100 ms		5 s
100 nm		500 μs		50 ms
***R_o_***	**c** **_∞_ ≈ ** **c_s_**	**Time** **to R = 0**	**c_∞_ ≈ c_s_**	**Time** **to R = 10 *R_o_***
10 μm	0.9999c_s_	3.5 h	1.0001	14 days
1 μm		125 s		3.4 h
100 nm		1.25 s		124 s

## Data Availability

The data presented in this study are available on request from the corresponding author.
